# The effective sedative dose of remimazolam for BIS <60 during general anesthesia induction between elderly and non-elderly patients-A randomized controlled trial

**DOI:** 10.3389/fphar.2025.1692105

**Published:** 2025-10-08

**Authors:** Jun-Li Zheng, Jie-Feng Sun, Qing Han, Si-Ren Shi, Jin Zhou, Wei-Long Wang, Xiao-Dong Huang, Zhen-Feng Zhou

**Affiliations:** ^1^ Department of Anesthesiology, The first People’s Hospital of Lin-ping District, Hangzhou, Zhejiang, China; ^2^ The Fourth School of Clinical Medicin, Zhejiang Chinese Medical University, Hangzhou, Zhejiang, China; ^3^ Department of Anesthesiology, Hangzhou Fu-yang Hospital of Traditional Chinese Medicine, Hangzhou, Zhejiang, China; ^4^ Department of Anesthesiology, Hangzhou Women’s Hospital (Hangzhou Maternity and Child Healthcare Hospital, Hangzhou First People’s Hospital Qianjiang New City Campus, Chinese Medical University), Hangzhou, Zhejiang, China

**Keywords:** elderly, non-elderly, remimazolam, sufentanil, effective dose

## Abstract

**Background:**

There is lack research about the effect of sufentanil on the effective dose of remimazolam during general anesthesia in both elderly and non-elderly patients scheduled for day surgery. This study was conducted to estimate the 95% effective dose (ED95) of remimazolam with low dose of sufentanil for BIS <60 during general anesthesia in both elderly and non-elderly patients scheduled for day surgery.

**Methods:**

Patients scheduled for elective gynecological day procedures were randomly allocated to one of four dosage groups receiving 0.1, 0.2, 0.3, or 0.4 mg/kg of remimazolam, with 25 participants per group. All patients received a concurrent intravenous dose of sufentanil (0.1 μg/kg) during anesthesia induction. Successful sedation was defined as achieving a BIS score of <60 within 5 minutes of remimazolam administration. The ED50 and ED95 of remimazolam for BIS <60 during general anesthesia induction were calculated. Secondary outcomes included the incidence of hypotension, respiratory depression, and adverse events.

**Results:**

In elderly patients, the estimated ED50 of remimazolam was determined to be 0.156 mg/kg, with a 95% confidence interval (CI) ranging from 0.110 to 0.190 mg/kg. For non-elderly individuals, the corresponding ED50 was 0.218 mg/kg (95% CI: 0.179–0.253 mg/kg). Additionally, the dose required to achieve 95% efficacy (ED95) was calculated as 0.336 mg/kg (95% CI: 0.286–0.437 mg/kg) in the elderly cohort and 0.418 mg/kg (95% CI: 0.361–0.528 mg/kg) in the non-elderly cohort. Hypotension occurred significantly more often in elderly patients (*P* < 0.05), but not for the incidence of bradycardia or respiratory depression between groups (*P* > 0.05).

**Conclusion:**

Under BIS monitoring during gynecological day surgery, the estimated ED95 of remimazolam with 0.1 μg/kg sufentanil was 0.336 mg/kg (95% CI: 0.286–0.437 mg/kg) for elderly patients and 0.418 mg/kg (95% CI: 0.361–0.528 mg/kg) for non-elderly patients.

**Clinical Trial Registration:**

This study is registered with ClinicalTrials.gov as ChiCTR2400091138.

## Introduction

Intravenous anesthetics achieve target sedation, they variably affect the respiratory and circulatory systems. Propofol and midazolam are the most widely used intravenous anesthetics (Chitilian et al., 2013). Propofol’s drawbacks include injection-site pain (Scott et al., 1988), cardiovascular depression, and respiratory depression (Khurmi et al., 2017). Midazolam’s propensity for peripheral tissue accumulation during prolonged or repeated administration delays post-infusion recovery (Barends et al., 2018).

An ideal sedative agent for anesthesia induction would exhibit rapid onset, short duration of action, hemodynamic stability, and organ-independent metabolism (Barends et al., 2018). Remimazolam, a novel ultra-short-acting benzodiazepine, exerts its effect through central GABA-A receptors and features rapid onset, predictable metabolism, minimal respiratory and circulatory suppression, and organ-independent elimination. Furthermore, its effects are reversible with flumazenil (Pesic et al., 2020; Sheng et al., 2020). These characteristics make remimazolam a promising agent for general anesthesia (Lu et al., 2022). The growing clinical demand for remimazolam in day surgery contexts. However, as it provides sedation without analgesia (Stöhr et al., 2021), it is commonly combined with opioid analgesics in day surgeries (Xu et al., 2023).

Sufentanil, a potent fentanyl derivative with high μ-opioid receptor affinity, offers effective analgesia, rapid systemic clearance, and a short recovery profile (Vendruscolo et al., 2018). The remimazolam–sufentanil combination is increasingly used for painless procedures such as gastrointestinal endoscopy due to its reliable anesthetic efficacy (Cao et al., 2022; Lyu et al., 2023). Recent evidence suggests that the effective dose of remimazolam required for anesthesia induction varies significantly among different age groups, particularly between elderly and younger patients (Huang et al., 2024; Huang et al., 2025).

This study aimed to determine the 95% effective dose (ED95) of remimazolam for BIS <60 during general anesthesia in elderly and non-elderly patients undergoing day surgery.

## Methods

### Study design

Following approval from the Ethics Committee of the First People’s Hospital of Lin-ping District of Hangzhou (IRB: 2024 Study No. 195) and acquisition of written informed consent, 200 patients scheduled for elective gynecological day procedures under general anesthesia were enrolled between 25 October 2024, and 31 January 2025.

### Inclusion and exclusion criteria

The inclusion criteria comprised patients aged ≥18 years, American Society of Anesthesiologists (ASA) classification I–III, scheduled for elective gynecological day procedures under general anesthesia, body mass index (BMI) of 18–30 kg/m^2^, willingness to participate and provide informed consent.

Exclusion criteria included: (1) emergency surgical requirements; (2) known hypersensitivity to remimazolam or contraindications to its use; (3) altered mental status or chronic pain requiring long-term sedative, or analgesics medication use; (4) use of sedatives, antiemetics, antipruritics, monoamine oxidase inhibitors, or antidepressants within 24 h preoperatively; (5) known difficult airway, respiratory insufficiency, or obstructive sleep apnea; (6) history of liver surgery, hepatorenal dysfunction, gastrointestinal ulcers, or coagulopathy; (7) active malignancy or significant cardiovascular/cerebrovascular disease; (8) participation in other clinical trials or deemed unsuitable by the investigators.

### Blinding and randomization

Randomization was performed by an independent researcher using computer-generated sequences (Microsoft Excel), and allocations were sealed in sequentially numbered opaque envelopes. Patients were stratified by age into two groups: Group O (elderly, ≥65 years, n = 100) and Group Y (non-elderly, 18–64 years, n = 100). Within each group, patients were randomly assigned to receive one of four remimazolam doses (0.1, 0.2, 0.3, or 0.4 mg/kg), forming subgroups O1–O4 and Y1–Y4 (25 patients per subgroup). Drug preparation was conducted by an independent nurse not involved in the study to maintain blinding of patients, anesthesiologists, surgeons, and data analysts. Remimazolam was diluted in 0.9% saline to a standardized volume of 20 mL, with concentration adjusted based on body weight.

### Anesthesia procedure

No sedatives were administered preoperatively. Standard intraoperative monitoring (Systolic Blood Pressure (SBP), Diastolic Blood Pressure (DBP), heart rate (HR) and Pulse Oximetry Oxygen Saturation (SpO_2_)) was initiated upon operating room arrival, supplemented by invasive monitoring when indicated. A disposable BIS sensor (Angel-1000A, Shenzhen Weihaokang Medical Devices Co., LTD., China) was used according to the manufacturer’s instructions. Prior to placement, the skin was cleaned with alcohol wipes to remove oils and ensure optimal adhesion. All patients received 2 L/min oxygen via facemask and intravenous sufentanil (injection, 50 μg/1 mL; Yichang Renfu Pharmaceutical Co., LTD., China, Batch No. H20054171) (0.1 μg/kg), followed by the assigned dose of remimazolam (injection, 36 mg; Jiangsu Hengrui Medicine Co., LTD., China, Batch No. H20190034) 1 minute later. Successful sedation was defined as achieving a BIS score of <60 within 5 minutes of remimazolam administration (Lysakowski et al., 2009). If BIS remained ≥60, propofol (injection, 200 mg/20 mL; Xi ‘an Libang Pharmaceutical Co., LTD., China, Batch No. H19990282) (1 mg/kg) was administered as a rescue sedative, repeated every 3 minutes as needed. Treated with airway support when SpO_2_ <90%.

Vital signs and minimum BIS values were recorded within 5 minutes following induction.

### Primary outcome

The primary outcome was the ED50 and ED95 of remimazolam for BIS <60 during general anesthesia induction.

Secondary outcomes included:

Respiratory depression (Schüttler et al., 2020): respiratory rate <8 breaths/min for >60 s and/or SpO_2_ <90%. Treated with airway support and high-flow oxygen.

Hypotension (Bijker et al., 2007): systolic BP < 90 mmHg or ≥30% decrease from baseline, managed with 0.5 mg metaraminol IV.

Bradycardia (Sessler et al., 2018): HR < 50 bpm for >30 s, treated with 0.5 mg atropine IV.

Hiccup: documented if present.

Baseline (before intravenous remimazolam injection) and follow-up measurements were recorded at 1-min intervals from T1 to T5 (first 5 min post-induction) to ensure comprehensive monitoring.

#### Sample size

Sample size was estimated using PASS 11 (NCSS, LLC) with Cochran-Armitage trend testing. Based on preliminary data indicating dose-dependent sedation success rates—0.1 mg/kg (20%), 0.2 mg/kg (50%), 0.3 mg/kg (60%), and 0.4 mg/kg (80%)—a minimum of 14 participants per group (N = 56) would provide 90% power (α = 0.05, two-tailed) to detect linear trends. To ensure statistical robustness and account for potential dropouts (estimated at 20%), a total of 200 participants (100 per age group) were enrolled.

### Statistical analysis

Normally distributed variables were presented as mean ± standard deviation (SD) and were analyzed using one-way ANOVA followed by Bonferroni correction, while non-normally distributed variables were reported as median (interquartile range) and analyzed using Kruskal–Wallis test and Dunn’s *post hoc* comparisons. Categorical data are expressed as n (%), and group comparisons were made using the chi-square test or Fisher’s exact test, as appropriate. ED50 and ED95 values with 95% CIs were estimated via Probit regression. A significance threshold of *P* < 0.05 was adopted (IBM SPSS Statistics 25.0 and GraphPad Prism 10.0).

## Results

### Baseline characteristics

**FIGURE 1:**
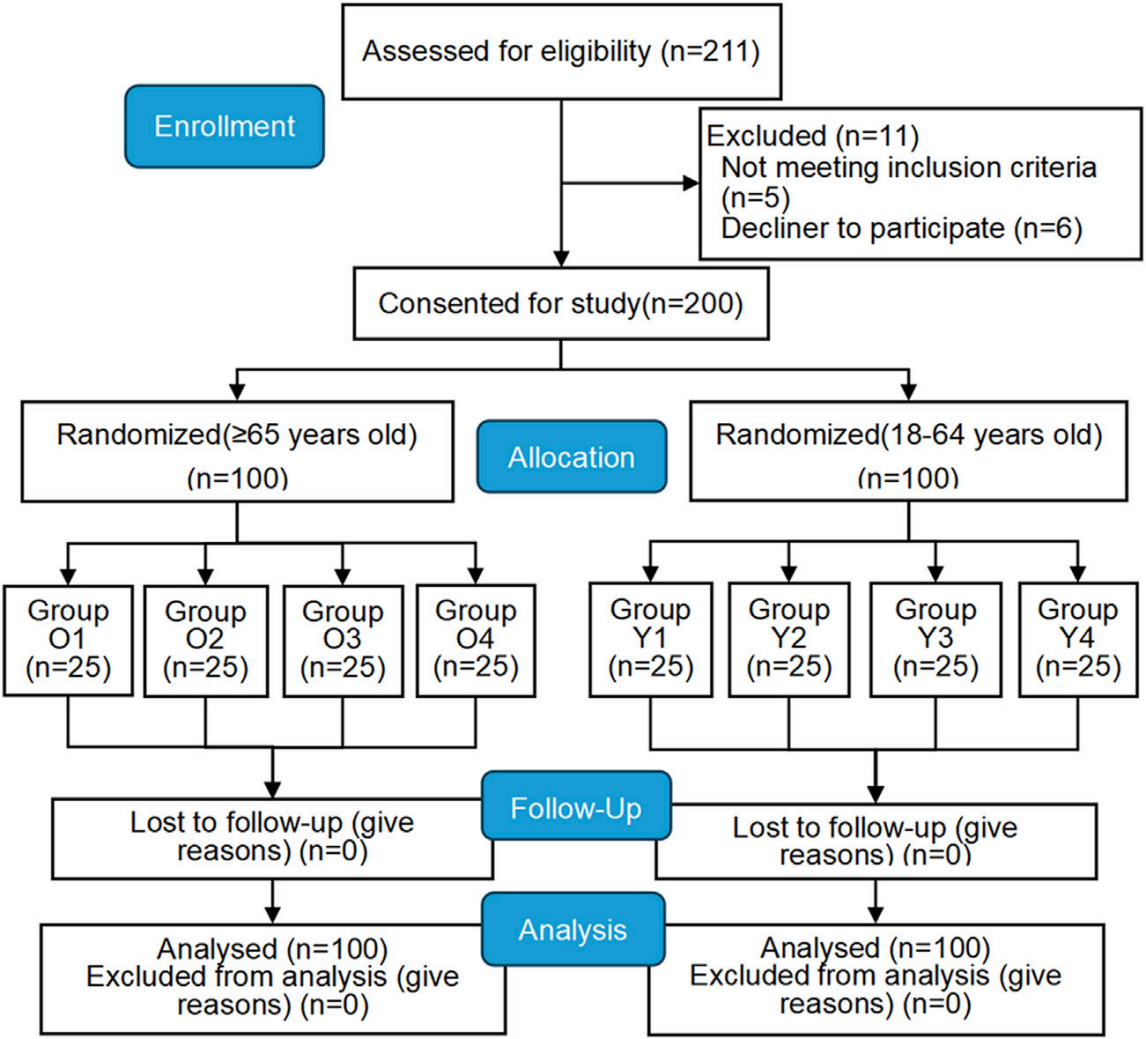
CONSORT flow diagram.

Among 211 patients initially screened, 11 were excluded due to failure to meet inclusion criteria, resulting in 200 participants (Figure 1).

Baseline demographic characteristics did not differ significantly between age groups (Table 1). No patients >80 years was enrolled.

**TABLE 1:** Characteristics of participants.

	Group O1	Group O2	Group O3	Group O4	*P-value*	Group Y1	Group Y2	Group Y3	Group Y4	*P-value*
Age.y (mean ± SD)	72.4±6.4	69.3±4.1	70.6±4.3	70.6±5.0	0.148	37.6±9.2	38.5±10.2	35.7±7.7	33.1±6.7	0.120
BMI, kg·m^−2 ^(mean ± SD)	23.9±2.4	24.5±3.0	22.4±2.7	24.1±2.6	0.295	20.8±2.1	21.9±2.4	21.5±1.8	22.1±2.2	0.124
BIS	95.6±3.5	94.3±4.6	96.8±2.7	96.8±2.3	0.339	96.7±3.0	97.2±2.7	96.8±2.7	96.4±3.2	0.456
ASA, n					>0.99					0.346
Ⅰ	0	0	0	0		5	7	4	2	
Ⅱ	25	25	25	25		20	18	21	23	
Ⅲ	0	0	0	0		0	0	0	0	
Coexistent disease, n(%)										
Hypertension	15(60)	13(52)	17(68)	18(72)	0.518	2(8)	3(12)	3(12)	1(4)	0.869
Diabetes	6(24)	5(20)	3(12)	5(20)	0.806	1(4)	0(0)	0(0)	0(0)	>0.99
COPD	2(8)	0(0)	1(4)	0(0)	0.613	0(0)	0(0)	0(0)	0(0)	>0.99
Type of operation, n(%)					0.519					0.600
Hysteroscopic	20(80)	22(88)	19(76)	23(92)		23(92)	22(88)	24(96)	20(80)	
Painless curettage	3(12)	2(8)	4(16)	0(0)		1(4)	2(8)	1(4)	4(16)	
Conization of cervix	2(8)	1(4)	2(8)	2(8)		1(4)	1(4)	0(0)	1(2)	

BMI, body mass index; BIS, bispectral index; ASA, American Society of Anesthesiologists.

### Remimazolam sedation dose (ED50 and ED95) for BIS <60 in elderly and non-elderly patients

**FIGURE 2:**
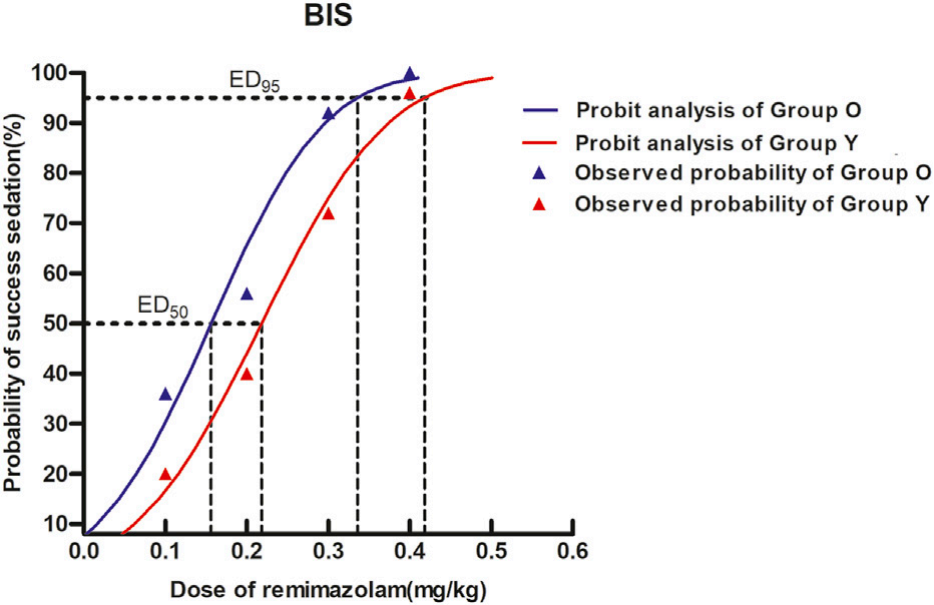
Dose–response curves for remimazolam in each group derived from probit analysis using BIS monitoring. Dashed line indicates the position of the estimate of ED95. The estimated ED95 of remimazolam were separately 0.336 mg/kg (95% CI: 0.286–0.437) and 0.418 mg/kg (95% CI: 0.361–0.528) in elderly and non-elderly patients.

In elderly patients, the estimated ED50 of remimazolam was determined to be 0.156 mg/kg, with a 95% confidence interval (CI) ranging from 0.110 to 0.190 mg/kg. For non-elderly individuals, the corresponding ED50 was 0.218 mg/kg (95% CI: 0.179–0.253 mg/kg). Additionally, the dose required to achieve 95% efficacy (ED95) was calculated as 0.336 mg/kg (95% CI: 0.286–0.437 mg/kg) in the elderly cohort and 0.418 mg/kg (95% CI: 0.361–0.528 mg/kg) in the non-elderly cohort, as illustrated in Table 2 and Figure 2.

**TABLE 2:** Trend comparison of the incidence of successful sedation and adverse events among the four dose groups.

	Older age group (n,%)				*P*-trend	Non-elderly group (n,%)				*P-trend*
	O1	O2	O3	O4		Y1	Y2	Y3	Y4	
Successful sedation	9 (36%)	14 (56%)	23 (92%)	25 (100%)	< 0.001	5 (20%)	10 (40%)	18 (72%)	24 (96%)	< 0.001
Hypotension	4 (16%)	9 (36%)	11 (44%)	12 (48%)	0.057	2 (8%)	2 (8%)	3 (12%)	5 (20%)	0.087
Bradycardia	0 (0%)	0 (0%)	1 (4%)	1 (4%)	0.106	0 (0%)	0 (0%)	0 (0%)	0 (0%)	>0.99
Respiratory depression	1 (4%)	7 (28%)	10 (40%)	12 (48%)	0.031	0 (0%)	7 (28%)	11 (44%)	12 (48%)	0.052
Hiccup	2 (8%)	0 (0%)	1 (4%)	1 (4%)	0.684	0 (0%)	1 (4%)	1 (4%)	2 (8%)	0.051

Data shown as number (%), P-trend < 0.05 considered linear trend.

Results of dose–response and dose-finding studies of remimazolam for BIS <60 were presented in S1 Supplementary Appendix 1.

### Adverse events

**FIGURE 3:**
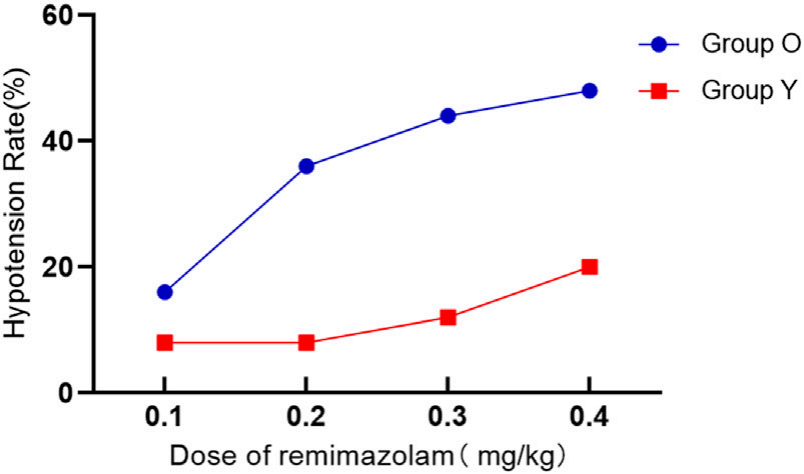
The incidence of hypotension in the elderly group compared to the non-elderly group.

Hypotension occurred significantly more often in elderly patients (*P* < 0.05), but not for the incidence of bradycardia, respiratory depression, or hiccups within the first 5 minutes following induction between groups (*P* > 0.05) (Table 3; Figure 3).

**TABLE 3:** Side effects during induction period between two groups.

	Older age group (n,%)	Non-elderly group (n,%)	*P-value*
Hypotension	36(36%)	12(12%)	0.001
Bradycardia	2(2%)	0(0%)	0.497
Respiratory depression	30(30%)	30(30%)	>0.99
Hiccup	4(4%)	4(4%)	>0.99

Data shown as number (%), *P* < 0.05 was deemed statistically significant.

The increase in the dose of remimazolam is associated with an increased risk of respiratory depression; as the dosage increases, the risk shows a rising trend (*P* = 0.031 for the elderly group and *P* = 0.052 for the non-elderly group). Among the four groups, the hypotension, bradycardia and hiccups had no linear relationship with the dose of ramazolam (*P* > 0.05) (Table 2).

### Haemodynamics

SBP, DBP, HR and SpO_2_ within 5 min after a single i.v. bolus of remimazolam and sufentanil were shown in S2 Supplementary Appendix 2.

SBP was different among the different dose of ramazolam in elderly patients over time (*P* < 0.05), but no significant differences were observed regard to DBP, HR and SpO_2_. For non-elderly patients, significant differences in SBP, HR and SpO_2_ were observed among the different dose of ramazolam (*P* < 0.05), but not for DBP.

## Discussion

This study demonstrated that, under bispectral index (BIS) monitoring and with concomitant opioid administration, the estimated ED95 of remimazolam for BIS <60 during general anesthesia induction was 0.418 mg/kg in non-elderly patients and 0.336 mg/kg in elderly patients undergoing gynecological day surgery-representing an approximate 19.6% reduction in the elderly cohort. Previous study have investigated the effect of age on the pharmacokinetics and pharmacodynamics of midazolam using the electroencephalogram (EEG) as a measure of the hypnotic-sedative effect, and suggest that the lower doses needed to reach sedation in the elderly subjects were attributable to a 50% decrease in EC50, not to changes in pharmacokinetics (Albrecht et al., 1999). Another research reported ED95 values of approximately 0.37 mg/kg of remimazolam for non-elderly and 0.25 mg/kg for elderly patients (Oh et al., 2022), although those studies did not incorporate opioid co-administration. The effective doses of this study were also higher than those reported in our previous studies on remimazolam-induced loss of consciousness in non-elderly and elderly patients (Schüttler et al., 2020; Bijker et al., 2007). This discrepancy may be attributed to differences in study design, statistical methodology, and the criteria used to define effective sedation. Specifically, our prior studies employed the Dixon sequential allocation method and utilized the MOAA/S (Modified Observer’s Assessment of Alertness/Sedation) scale to assess loss of consciousness. Importantly, the present study examined the use of a single low dose of sufentanil, commonly administered in clinical contexts such as ambulatory surgery or painless diagnostic procedures, rather than the higher doses typically used for anesthesia induction.

In clinical settings, general anesthesia induction is commonly performed using a combination of sedatives and opioids due to their synergistic effects (Catalani et al., 2022). Our prior work has shown that fentanyl can reduce the effective dose of remimazolam by 30%–50% (Schüttler et al., 2020; Bijker et al., 2007). In this study, sufentanil was used, supporting a multimodal approach to optimize sedation while minimizing adverse events.

Several studies have investigated the effective dose of remimazolam for anesthesia induction. One study reported that a dose of 0.3 mg/kg successfully induced anesthesia in 94% of patients, with minimal impact on hemodynamic stability (Dai et al., 2021). In this study, effective sedation was alternatively defined in prior studies by a MOAA/S score ≤1. Another study, utilizing the up-and-down sequential allocation method, reported the ED95 of remimazolam as 0.118 mg/kg for patients aged 60–69 years and 0.090 mg/kg for those aged 70–85 years, with efficacy defined by a MOAA/S score of 0 (Liu et al., 2022). Additionally, Dongwoo Chae et al. (Chae et al., 2022) proposed age-specific dosing recommended remimazolam dosages of 0.25–0.33 mg/kg for patients under 40 years, 0.19–0.25 mg/kg for those aged 60–80 years, and 0.14–0.19 mg/kg for individuals older than 80 years, again using a MOAA/S score ≤1 as the criterion for effective sedation. Discrepancies between these findings and our study may be attributed to variations in the definition of successful sedation, differences in statistical methodologies, and the influence of concomitant medications. A detailed comparison of our findings with prior studies is available in Supplementary Appendix S1. Notably, in previous studies, the effective sedative dose was assessed within 1 min of drug administration, whereas our study assessed sedative dose using BIS within 5 min after induction, a longer observation window than in many prior studies, allowing for more robust evaluation of remimazolam’s direct effects during the induction phase-typically unaffected by surgical stimuli or blood loss.

Both BIS and MOAA/S have also been widely utilized in research evaluating the effective dosing of remimazolam (Dai et al., 2021; Liu et al., 2022; Suzuki et al., 2023; Bae et al., 2024; Zhao et al., 2023). BIS monitoring is a well-established method for evaluating sedation depth (Yang et al., 2024), while MOAA/S scoring is also frequently used due to its stability and responsiveness (Bae et al., 2024). However, emerging evidence suggests that BIS may also provide a reliable, objective measure of sedation depth during remimazolam use (Bae et al., 2024; Zhao et al., 2023). Furthermore, opioids may alter cortical activity patterns, raising questions about the reliability of MOAA/S scores in this context (Vakkuri et al., 2004). Our BIS-based approach mitigates these limitations, offering a more objective measure of sedation. BIS values were usually maintained between 40 and 60 during anesthesia maintenance. This was also an objective indicator for us to judge the depth of anesthesia in patients in clinical practice, and our medication adjustments wer based on this, so BIS <60 was the efficacy endpoint in this trial.

Remimazolam has demonstrated favorable cardiovascular safety compared to propofol, particularly in frail patients with ASA I–III status (Doi et al., 2020a; Doi et al., 2020b). Nonetheless, our study revealed a higher incidence of hypotension in elderly patients, possibly influenced by comorbidities such as hypertension (prevalence: 63%). Remimazolam activating the central GABA_a_ receptors can inhibit the vasomotor center and further reduce sympathetic output, especially in elderly patients (Ju et al., 2024). When used in combination with sufentanil, it can synergistically inhibit sympathetic activity and increase the risk of hypotension (Barbosa et al., 2024). Even at ED50 (0.156 mg/kg), more than one-quarter of elderly patients experienced significant hypotension, necessitating vasopressor use and slow titration administration. This contrasts with prior findings in healthier populations (Dai et al., 2021).

The incidence of respiratory depression was not significant difference between age groups, although during the induction of anesthesia, a dose-dependent respiratory depression phenomenon occurred, especially in the elderly group of patients. At 0.4 mg/kg, nearly half of all patients experienced respiratory depression, though none had SpO_2_ levels below 85%. The absence of artificial airway support may have contributed to this trend, possibly due to transient upper airway obstruction. At the estimated ED95 of remimazolam in elderly patients and non-elderly patients undergoing gynecological day surgery, the incidence rates of hypotension were 44% and 20% respectively, and the incidence rates of respiratory depression were 40% and 48% respectively, which suggested preparing medication and airway support items given these safety concerns. Hiccups were infrequent and comparable across groups.

## Limitations

This study has several limitations. First, the study population (mainly ASA I–II patients undergoing gynecological day surgery) limits extrapolation to higher-risk surgical populations (e.g., ASA III or above). Second, artificial airway devices (e.g., oropharyngeal airways, laryngeal masks) were not used, leaving open the possibility of undetected retro lingual obstruction contributing to hypoxemia. Thirdly, the pharmacokinetic (PK) or plasma concentration measurements of remimazolam was lack. The study conclusions rely solely on BIS-based endpoints and future studies should emphasize PK/PD validation. Lastly, while our analysis focused on sedative dose following induction, we did not evaluate intraoperative anesthetic requirements or postoperative recovery metrics. Future studies should include a comprehensive perioperative assessment.

## Conclusion

In patients undergoing gynecological day surgery under BIS monitoring, the ED95 of remimazolam combined with sufentanil for general anesthesia induction was significantly lower in elderly patients (0.336 mg/kg) compared to non-elderly patients (0.418 mg/kg). Elderly patients exhibited a higher incidence of hypotension, highlighting the need for careful hemodynamic monitoring and vasopressor support during induction. These findings support the age-based adjustment of remimazolam dosing to enhance safety and efficacy in clinical anesthesia practice.

## Data Availability

The original contributions presented in the study are included in the article/Supplementary Material, further inquiries can be directed to the corresponding author.
