# Supplementary material for: The effective sedative dose of remimazolam for BIS <60 during general anesthesia induction between elderly and non-elderly patients-A randomized controlled trial
Source: Front Pharmacol. 2025 Oct 8;16:1692105. doi: 10.3389/fphar.2025.1692105 (PMC12540430; doi:10.3389/fphar.2025.1692105)
Supplement: Supplementary file 1 [file Supplementaryfile1.doc]

**S1 Supplemental Appendix 1. Results of Dose–Response and Dose-Finding Studies of remimazolam for induction of general anesthesia.**

|  | Effective dose of Remimazolam (mg/kg) | Combined adjuvant drug | Age (y) | Outcome (Response) | Methodology |
| --- | --- | --- | --- | --- | --- |
| Dai G 2021^[22]^ | 0.3(94% efficacy) |  | 18-65 | MOAA/S≤1 | Up and down |
| MiaoLiuet 2022^[23]^ | ED_95_:0.118(0.103–0.649) |  | 60–69 | MOAA/S=0 | Up and down |
|  | ED_95_:0.090(0.075–0.199) |  | 70–85 | MOAA/S=0 | Up and down |
| Dongwoo Chaeet2022^[24]^ | ED_95_:0.25-0.33 |  | <40 | MOAA/S≤1 | Weibull and log-logistic models |
|  | ED_95_:0.19-0.25 |  | 60-80 | MOAA/S≤1 | Weibull and log-logistic models |
|  | ED_95_:0.14-0.19 |  | >80 | MOAA/S≤1 | Weibull and log-logistic models |
| Xiaodong Huang et al2024^[14]^ | ED_95_:0.117 (0.095–0.194) | fentanil(1µg/kg) | 65–80 | MOAA/S=0 | Up and down |
|  | ED_95_:0.161 (0.130-0.280) |  | 65-80 | MOAA/S=0 | Up and down |
| Xiaodong Huang et al2025^[15]^ | ED_95_:0.235(0.189-0.316) | fentanil(1µg/kg) | 18-60 | MOAA/S=0 | Random allocation  and probit analysis |
|  | ED_95_:0.453(0.364-0.640) |  | 18-60 | MOAA/S=0 | Random allocation  and probit analysis |
| Junli Zheng et al(This study) | ED_95_:0.418(0.361-0.528) | Sufentanil (0.1μg/kg) | 18-64 | BIS<60 | Random allocation  and probit analysis |
|  | ED_95_:0.336(0.286-0.437) | Sufentanil (0.1μg/kg) | ≥65 | BIS<60 | Random allocation  and probit analysis |

Dose values are in Milligrams per kilogram with 95% confidence intervals in parentheses. Definitions for effective dose of remimazolam varied among studies; see text for explanation
