# Supplementary figures and images for: The effective sedative dose of remimazolam for BIS <60 during general anesthesia induction between elderly and non-elderly patients-A randomized controlled trial
Source: Front Pharmacol. 2025 Oct 8;16:1692105. doi: 10.3389/fphar.2025.1692105 (PMC12540430; doi:10.3389/fphar.2025.1692105)

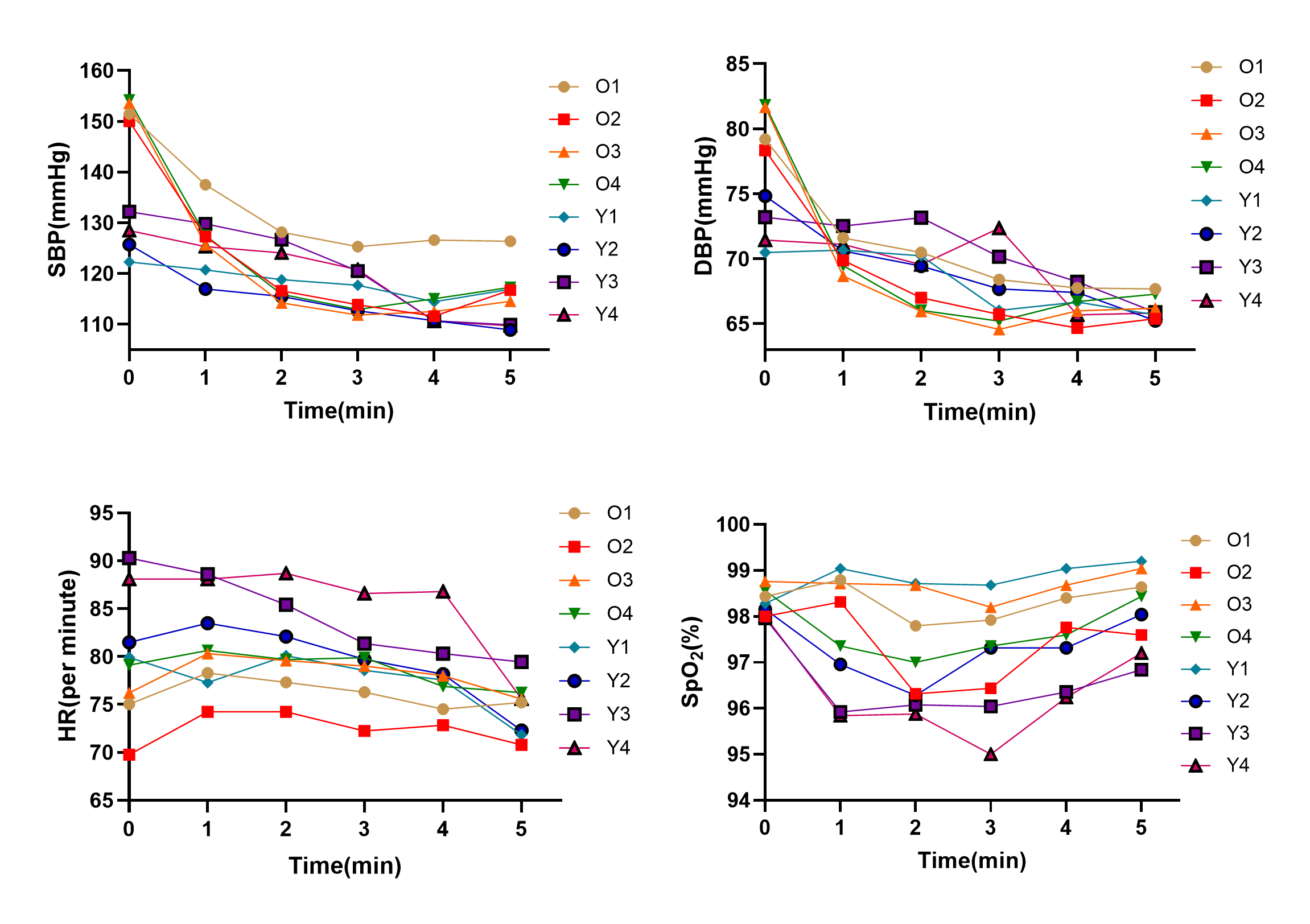

Supplement: Supplementary file 2 [file Supplementaryfile2.tif]
